# Giant intra-abdominal lymphatic malformation arising from the cisterna chyli-thoracic duct region in a five-year-old boy: a case report

**DOI:** 10.1093/jscr/rjag1002

**Published:** 2026-10-31

**Authors:** Nikolaos Baltogiannis, Fotini Fili, Melina E Baltogianni

**Affiliations:** Pediatric Surgery Department, Children’s Hospital “Athens Medical Group”, 5–7 Distomou Street, Marousi, Athens, Attica 15125, Greece; Pediatric Surgery Department, Children’s Hospital “Athens Medical Group”, 5–7 Distomou Street, Marousi, Athens, Attica 15125, Greece; Department of Obstetrics and Gynecology, General Hospital of Patras “Agios Andreas”, 37 Kalavryton Street, Patras, Achaia 26335, Greece

**Keywords:** lymphatic malformation, cystic lymphangioma, cisterna chyli, thoracic duct, pediatric surgery

## Abstract

Lymphatic malformations are rare congenital vascular anomalies, and intra-abdominal involvement is uncommon. We report a 5-year-old boy with progressive abdominal distension for more than 6 months and intermittent abdominal pain. Ultrasonography and magnetic resonance imaging demonstrated a giant multiloculated cystic lesion measuring approximately 21 × 13 × 23 cm, occupying most of the abdominal cavity and markedly displacing the abdominal viscera. Because of the lesion size and severely restricted intra-abdominal working space, open surgical excision was performed. Intraoperatively, part of the malformation arose from the cisterna chyli-thoracic duct region and contained inspissated chylous material. Complete excision was achieved without bowel resection. Histopathology and immunohistochemistry confirmed cystic lymphangioma, while a separately submitted lymphatic structure was compatible with the thoracic duct. Recovery was uneventful, with no recurrence during 3 years of follow-up.

## Introduction

Lymphatic malformations (LMs), historically termed lymphangiomas, are congenital vascular malformations resulting from abnormal development of the lymphatic system and are classified as vascular malformations rather than true neoplasms [1]. They usually become apparent during early childhood and most frequently involve superficial cervicofacial regions, whereas intra-abdominal lesions are uncommon and may arise from the mesentery, omentum, retroperitoneum, gastrointestinal tract, or visceral organs [2].

Intra-abdominal LMs may remain clinically silent until considerable enlargement produces abdominal distension, pain, or complications related to mass effect [2, 3]. We report a giant intra-abdominal LM in a 5-year-old boy arising from the cisterna chyli-thoracic duct region.

## Case report

A 5-year-old boy was referred with progressive abdominal distension. His parents had noticed an unexplained increase in abdominal girth for more than 6 months. During the preceding 2 months, he had also experienced intermittent, poorly localized abdominal pain, without vomiting or constipation.

Initial ultrasonography performed at the referring institution demonstrated a large, predominantly fluid-filled, septated intra-abdominal lesion measuring approximately 20 × 13 cm. The patient was subsequently referred for definitive management.

Abdominal ultrasonography and contrast-enhanced magnetic resonance imaging (MRI) were performed. MRI demonstrated a giant cystic lesion measuring approximately 21 × 13 × 23 cm, occupying almost the entire abdominal cavity. Internal septa showed mild contrast enhancement. The lesion caused extensive displacement of the abdominal viscera, including posterior displacement of the spleen, adrenal glands, pancreas, and kidneys. Most of the small bowel was displaced toward the right upper quadrant, while the colon and left hepatic lobe were markedly displaced and distorted. Although imaging strongly favored a giant cystic LM, its precise anatomical origin could not be confidently established preoperatively.

Given the enormous lesion size, marked abdominal distension (Fig. 1), and severely restricted intra-abdominal working space, an open surgical approach was selected. At laparotomy, a giant multiloculated cystic lesion occupying most of the abdominal cavity was encountered (Fig. 2). A component of the malformation arose from the region of the cisterna chyli and origin of the thoracic duct, where inspissated chylous material was identified (Fig. 3). Complete excision of the lesion and involved lymphatic tissue was achieved without bowel resection.

**Figure 1:**
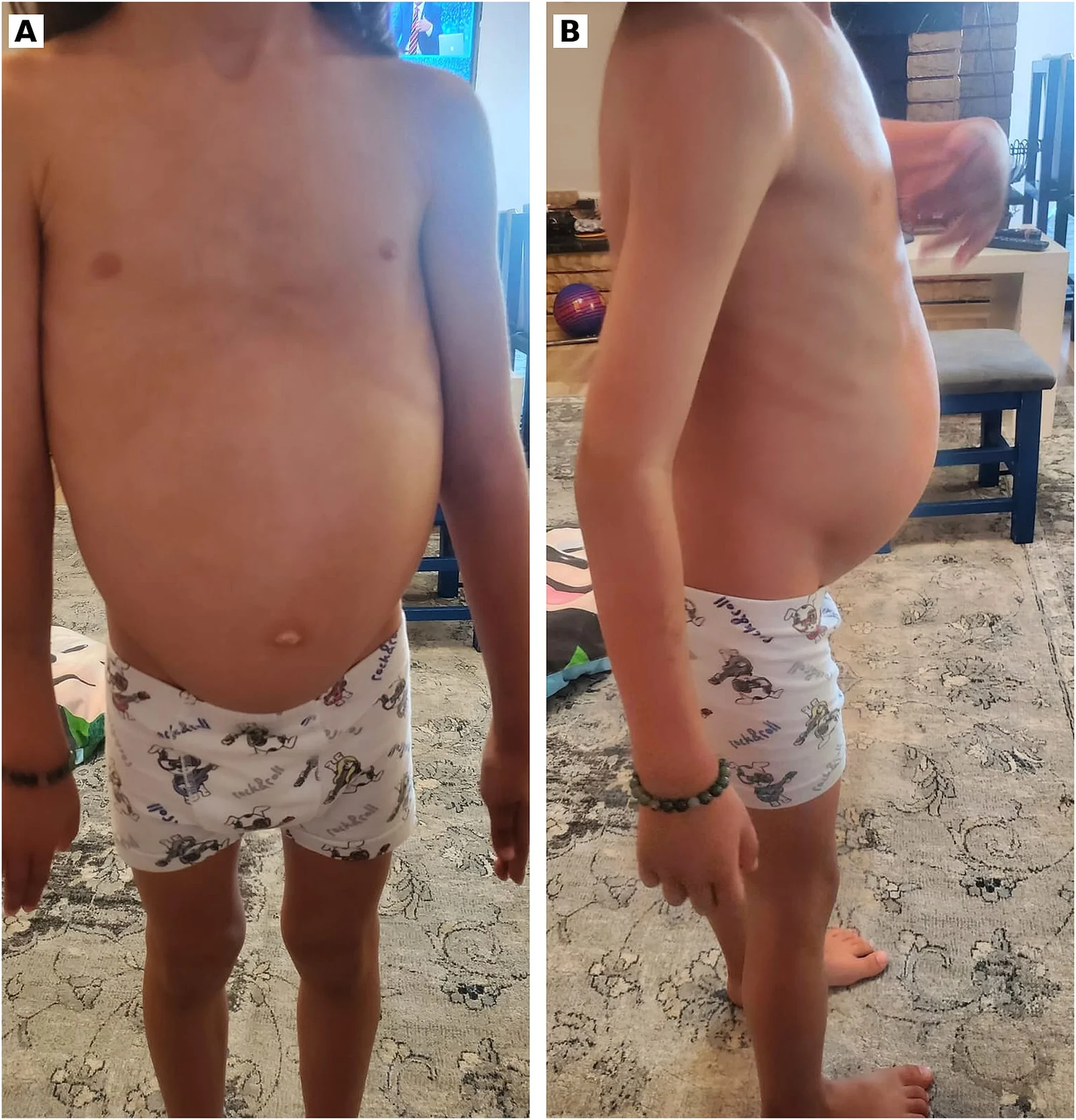
Preoperative clinical appearance. Frontal (A) and lateral (B) views demonstrating marked abdominal distension caused by the giant intra-abdominal lymphatic malformation.

**Figure 2:**
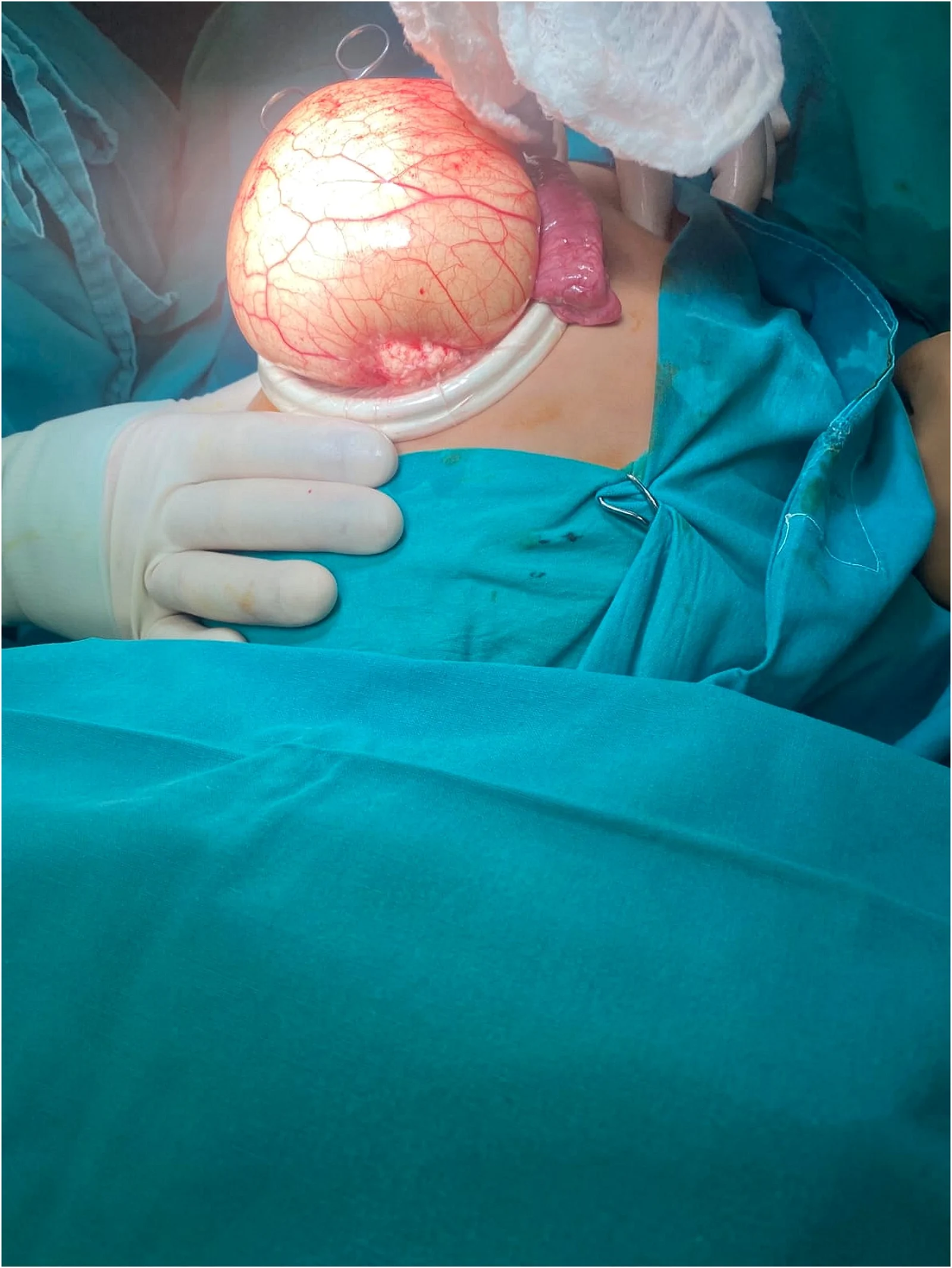
Intraoperative appearance of the lymphatic malformation. Intraoperative view immediately after laparotomy demonstrating the giant cystic lymphatic malformation occupying the abdominal cavity.

**Figure 3:**
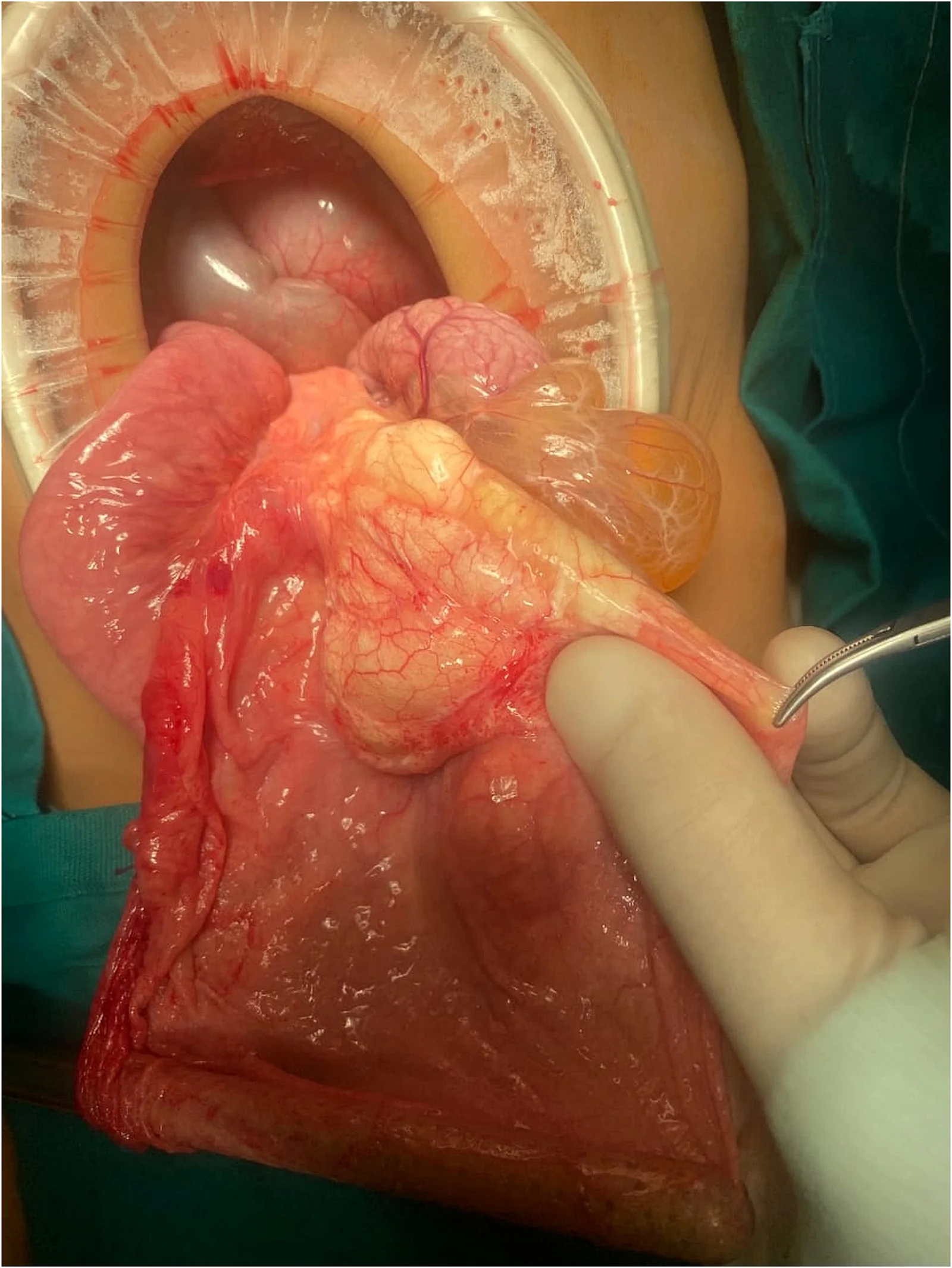
Intraoperative identification of the central lymphatic component. Intraoperative view demonstrating the component of the lymphatic malformation associated with the cisterna chyli–thoracic duct region.

Gross examination demonstrated a multiloculated cystic lesion containing characteristic white-milky fluid. Cytological examination revealed histiocytes and lymphocytes without evidence of malignancy. Histologically, variably sized cystic spaces were lined by flattened cells without cytologic atypia, pleomorphism, or evident mitotic activity. The cyst walls contained fibrous and fibroadipose tissue, smooth muscle components, and chronic inflammatory infiltrates. Immunohistochemical evaluation, including CD31 and D2-40, supported lymphatic endothelial differentiation and confirmed the diagnosis of cystic (cavernous) lymphangioma. A separately submitted specimen demonstrated a segment of a large lymphatic vessel morphologically compatible with the thoracic duct, concordant with the intraoperative findings.

The postoperative course was uneventful. At 3 years of follow-up, the patient remained clinically well with no evidence of recurrence.

## Discussion

Intra-abdominal LMs are uncommon in children and may remain clinically silent until enlargement causes abdominal pain, distension, or compression of adjacent structures. In a recent pediatric series, the median age at diagnosis was 6.6 years, and complete macroscopic resection was achieved in 91% of surgically explored patients [4]. A giant abdominal LM previously reported in the *Journal of Surgical Case Reports* similarly occupied almost the entire abdominal cavity, illustrating the difficulty of determining the exact anatomical origin of very large lesions preoperatively [5].

The distinctive feature of our case was involvement of the central lymphatic pathway. The cisterna chyli is a variable abdominal lymphatic structure from which the thoracic duct originates [6]. Thoracic duct cysts are exceptionally rare and may occur anywhere along the course of the duct [7]. In our patient, part of the malformation arose from the cisterna chyli-thoracic duct region and contained inspissated chylous material. This operative finding was supported by the characteristic white-milky cystic content and histopathological identification of a separately excised large lymphatic vessel compatible with the thoracic duct. A previously reported thoracic duct cyst extending to the cisterna chyli similarly contained milky-white fluid and demonstrated smooth muscle within the cyst wall [8]. Because the main specimen in our patient demonstrated an extensive multicystic LM rather than an isolated ductal cyst, we consider the lesion best classified as a giant LM arising from the cisterna chyli-thoracic duct region.

Management should be individualized according to symptoms, size, anatomical extent, and involvement of adjacent structures. Surgical resection provides favorable outcomes for localized and technically resectable intra-abdominal LMs [9]. Although minimally invasive excision is feasible in selected children, very large lesions may compromise visualization and operative working space and can necessitate conversion to laparotomy [10]. In our patient, the 23-cm lesion occupied almost the entire abdominal cavity; primary laparotomy allowed direct identification of its central lymphatic origin and complete excision without bowel resection.

Complete macroscopic resection appears to provide excellent long-term control for localized intraperitoneal LMs [4, 9]. The absence of recurrence at 3 years supports complete excision as definitive treatment when it can be achieved safely. Giant abdominal LMs may obscure their true anatomical origin on preoperative imaging; careful intraoperative assessment of possible involvement of the cisterna chyli and thoracic duct may therefore be essential for complete and safe resection.

## Data Availability

All data relevant to this case are included in the article. Additional de-identified information may be available from the corresponding author upon reasonable request.

## References

[1] Kunimoto K, Yamamoto Y, Jinnin M. ISSVA classification of vascular anomalies and molecular biology. Int J Mol Sci 2022;23:2358. 10.3390/ijms23042358 PMC8876303 35216474

[2] Lal A, Gupta P, Singhal M et al. Abdominal lymphatic malformation: Spectrum of imaging findings. Indian J Radiol Imaging 2016;26:423–8. 10.4103/0971-3026.195777 PMC5201068 28104932

[3] Francavilla ML, White CL, Oliveri B et al. Intraabdominal lymphatic malformations: pearls and pitfalls of diagnosis and differential diagnoses in pediatric patients. AJR Am J Roentgenol 2017;208:637–49. 10.2214/AJR.16.17008 28004970

[4] Hyvönen H, Aronniemi J, Salminen P et al. Operative management of intra-abdominal lymphatic malformations in children: a single tertiary center experience. J Pediatr Surg 2024;59:1875–8. 10.1016/j.jpedsurg.2024.05.011 38890018

[5] Gasparella P, Singer G, Castellani C et al. Giant lymphatic malformation causing abdominal compartment syndrome in a neonate: a rare surgical emergency. J Surg Case Rep 2020;2020:rjaa252. 10.1093/jscr/rjaa252 PMC7449550 32874540

[6] Chandra R, Murphy T, Jordan KG et al. “Mega” cisterna chyli: a case report and review of the literature. Cureus 2023;15:e34111. 10.7759/cureus.34111 PMC9946758 36843809

[7] Halliday LJ, Sharma AK. Multiple thoracic duct cysts: an unusual CT finding. J Surg Case Rep 2015;2015:rjv056. 10.1093/jscr/rjv056 PMC4423054 25952956

[8] Miyamoto H, Lee S, Ishidate T et al. A case of a thoracic duct cyst extending from the mediastinum to the cisterna chyli resected using bilateral thoracoscopic surgery in the prone position. Surg Case Rep 2023;9:171. 10.1186/s40792-023-01740-6 PMC10519914 37747542

[9] Elbaaly H, Piché N, Rypens F et al. Intra-abdominal lymphatic malformation management in light of the updated International Society for the Study of Vascular Anomalies classification. Pediatr Radiol 2021;51:760–72. 10.1007/s00247-020-04930-8 33559726

[10] Liu Q, Fu J, Yu Q et al. Laparoscopic surgery of intra-abdominal lymphatic malformation in children. Exp Ther Med 2022;24:581. 10.3892/etm.2022.11519 PMC9353511 35949324

